# Lack of Outer Membrane Protein A Enhances the Release of Outer Membrane Vesicles and Survival of *Vibrio cholerae* and Suppresses Viability of *Acanthamoeba castellanii*

**DOI:** 10.1155/2014/610190

**Published:** 2014-04-01

**Authors:** Soni Priya Valeru, Salah Shanan, Haifa Alossimi, Amir Saeed, Gunnar Sandström, Hadi Abd

**Affiliations:** ^1^Division of Clinical Microbiology F 82, Department of Laboratory Medicine, Karolinska Institute, Karolinska University Hospital, Huddinge, 141 86 Stockholm, Sweden; ^2^Faculty of Medical Laboratory Sciences, University of Medical Sciences and Technology, 11111 Khartoum, Sudan

## Abstract

*Vibrio cholerae*, the causative agent of the diarrhoeal disease cholera, survives in aquatic environments. The bacterium has developed a survival strategy to grow and survive inside *Acanthamoeba castellanii*. It has been shown that *V. cholerae* expresses outer membrane proteins as virulence factors playing a role in the adherence to interacted host cells. This study examined the role of outer membrane protein A (OmpA) and outer membrane vesicles (OMVs) in survival of *V. cholerae* alone and during its interaction with *A. castellanii*. The results showed that an *OmpA* mutant of *V. cholerae* survived longer than wild-type *V. cholerae* when cultivated alone. Cocultivation with *A. castellanii* enhanced the survival of both bacterial strains and *OmpA* protein exhibited no effect on attachment, engulfment, and survival inside the amoebae. However, cocultivation of the *OmpA* mutant of *V. cholerae* decreased the viability of *A. castellanii* and this bacterial strain released more OMVs than wild-type *V. cholerae*. Surprisingly, treatment of amoeba cells with OMVs isolated from the *OmpA* mutant significantly decreased viable counts of the amoeba cells. In conclusion, the results might highlight a regulating rule for *OmpA* in survival of *V. cholerae* and OMVs as a potent virulence factor for this bacterium towards eukaryotes in the environment.

## 1. Background

*Vibrio cholerae* is a curved, rod-shaped, Gram-negative bacterium that causes the severe diarrhoeal disease cholera. Its natural ecosystem includes aquatic environments in endemic locations. Two major virulence factors of* V. cholerae *are cholera toxin (CT) and an intestinal colonization factor known as the toxin coregulated pilus (TCP). Recent studies show that* V. cholerae* has developed a survival strategy to grow and survive inside the free-living amoeba* Acanthamoeba castellanii* [1–3].

*Vibrio cholerae* responds to environmental changes by altering the protein composition of its outer membrane (OM). This OM is composed of protein and lipopolysaccharide (LPS) [4, 5]. However, it has been found that OmpA of* V. cholerae *shares 47.8% similarity with that of* Escherichia coli*. OmpA is a *β*-barrel protein in the membrane and is highly conserved among Gram-negative bacteria [6]. It is expressed to very high levels and is tightly regulated at the posttranscriptional level. It can function as an adhesin and invasin, participate in biofilm formation, act as both an immune target and evasin, and serve as a receptor for several bacteriophages [7, 8]. It has been shown that* E. coli* utilizes OmpA for adhesion to Hela epithelial cells and Caco-2 colonic epithelial cells [9].

OMVs are produced by most Gram-negative bacteria, including* Vibrio* species [10]. The vesicles contain outer membrane proteins, lipopolysaccharides, and phospholipids and, as they are being released from the surface, the vesicles entrap some of the underlying periplasm. They can deliver toxins and other virulence factors to the host at relatively high concentrations, without requiring close contact between the bacterial and target human cells, and are believed to represent a key factor in effecting an inflammatory response in the host to bacterial pathogens [11–16].

OMVs are released by Gram-negative bacteria as a novel stress response [14, 17], whereas outer membrane proteins (Omp) play a major role in adherence to mucosal membrane in the small intestine and possible protective antigens [14]. The aim of this study was thus to examine the role and influence of OmpA and OMVs in the survival of* V. cholerae* alone and its interaction with* A. castellanii*.

## 2. Materials and Methods

### 2.1. Microorganisms, Culture Media, and Growth Conditions

The bacterial strains used in this study were wild-type* Vibrio cholerae* strain A1552 O1 El Tor Inaba [18] and its* OmpA* mutant by internal in-frame deletion of* OmpA* gene, kindly provided by Dr. S. N. Wai, University of Umeå. It has been proven that the* OmpA* mutant bacterium fails to produce OmpA protein, unlike the wild-type strain [7].* Acanthamoeba castellanii* was obtained from the American Type Culture Collection (ATCC 30234), Manassas, VA, USA.

*V. cholerae* strains were stored frozen in Luria-Bertani (LB) medium with 15% glycerol at −80°C. Both bacterial strains were grown overnight at 37°C on LB plates and thereafter in LB broth, with shaking to an absorbance_600_ of 0.6.* A. castellanii* was grown without shaking at 30°C to a final concentration of 10^6^/mL in ATCC medium number 712.

### 2.2. Cocultivation

The cocultivation assay was based on a method presented previously [1]. Axenically maintained amoebae were grown at 30°C to a final concentration of 2 × 10^6^ cell/mL in ATCC medium, as described above. Cocultivations of* V. cholerae* with* A. castellanii *were incubated in NUNC tissue culture flasks (75 cm^2^) purchased from VWR International (Stockholm, Sweden). Each flask contained 50 mL ATCC medium 712 containing* A. castellanii* at a concentration of 2 × 10^5^ cell/mL and the particular* V. cholerae *species at a concentration of 2 × 10^6^ cell/mL. Control flasks containing bacteria or amoebae only were prepared in the same way and with the same initial concentration as the coculture flasks. All flasks were prepared in triplicate and incubated at 30°C. Samples were taken and plated on blood agar plates regularly to study the growth and survival of* V. cholerae*.

### 2.3. Isolation of Outer Membrane Vesicles and Estimation of Protein Concentration

OMVs were isolated by ultracentrifugation, as described previously [16].* V. cholerae* strains were grown in broth culture to late exponential phase. Broth cultures were then centrifuged at 8,000 g (30 min, 4°C) in a JA-25.50 rotor (Beckman Instruments Inc.). Filtered (0.22 *μ*m; Millipore) supernatants were centrifuged at 85,000 g (2 h, 4°C) in a 70 Ti rotor (Beckman Instruments Inc.) to collect OMVs. Pellets were washed twice with PBS, suspended in PBS to a total volume of 500 *μ*L, and used as the OMVs preparation. Concentration of total protein in the OM vesicles was measured spectrophotometrically by the Bradford assay (Bio-Rad).

The effect of outer membrane vesicles on the viability of* A. castellanii* was examined by incubation of 50 *μ*L amoeba cell suspension containing 10^6^ cell/mL with 50 *μ*L OM vesicle preparation from each bacterial strain or with 50 *μ*L BPS for controls. Triplicate experiments were performed and the viability of amoebae was examined after 2 hours by viable count utilizing erythromycin B stain (ATCC).

### 2.4. Bacterial Adherence

Cocultures of the* V. cholerae* A1552 and* OmpA* mutant strains with* A. castellanii* were incubated in 75 cm^2^ cell culture flasks containing 50 mL ATCC medium number 712 with an initial concentration of 10^5^ cells of* A. castellanii*/mL and 10^6^ cells of each* V. cholerae* strain/mL. The flasks were incubated without shaking at 30°C and samples were withdrawn after 1 h to determine the percentage of bacteria adhering to the amoeba cells. This was done by dividing the number of amoebae with adhered bacteria by the total number of amoebae with and without adhered bacteria and multiplying by 100.

### 2.5. Bacterial Uptake, Growth, and Intracellular Survival

The ability of* A. castellanii* to take up* V. cholerae* A1552 strains and the effect of the* OmpA* mutant on uptake and intracellular growth of the bacterial strains were examined by comparing the interactions of wild-type A1552 and* OmpA* mutant with the amoebae.

Cocultures of each bacterial strain with* A. castellanii* were incubated in 75-mL cell culture flasks containing 50 mL ATCC medium number 712 with an initial concentration of 10^5^ cells of* A. castellanii*/mL and 10^6^ cells of each bacterial strain/mL. The flasks were incubated without shaking at 30°C for 2 h. Each cell suspension was centrifuged for 10 min at 300 g in a Labofuge GL centrifuge (VWR International) and washed six times in PBS to remove nonadhered extracellular* V. cholerae*. The pellets were resuspended in 1 mL PBS and incubated with 500 mg gentamicin/mL for 1 h at room temperature. The samples were then diluted in 9 mL PBS and centrifuged for 10 min at 300 g. Each pellet was resuspended in a volume of 50 mL ATCC medium in a 75 cm^2^ culture flask to analyse uptake, intracellular growth, and survival of* V. cholerae* strains. A 1 mL portion from each flask was centrifuged for 10 min at 300 g and each pellet was diluted twofold with 0.1% sodium deoxycholate to permeabilize the amoeba cells. A series of 10-fold dilutions of the sample from 10^1^ to 10^10^ was prepared and spread on blood agar plates. All plates were incubated overnight at 37°C, and viable counts were performed for the engulfed bacteria. The reculture flasks were incubated without shaking at 30°C to investigate the intracellular growth and survival of* V. cholerae* strains using a gentamicin assay and by viable counts for 15 days.

## 3. Results

### 3.1. Adherence, Uptake, and Intracellular Growth

To estimate adherence of* V. cholerae* wild-type and* OmpA* mutant strain to amoeba cells, the percentage of each bacterial strain adhering to* A. castellanii* was determined and found to be 83.3 ± 2.1% and 73.3 ± 3.5%, respectively. The difference in adherence between the wild-type and* OmpA* mutant of* V. cholerae* was not statistically significant (*t*-test, *P* = 0.29).

**Figure 1:**
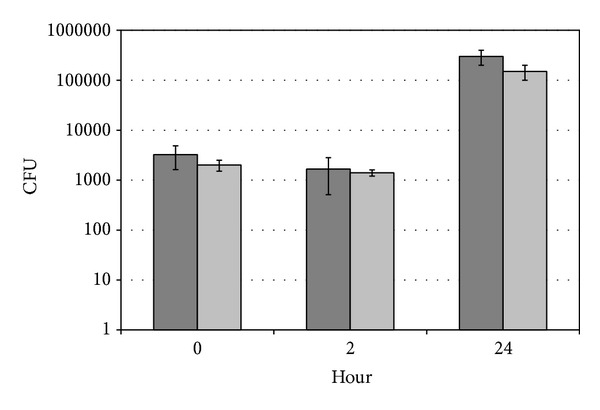
Uptake, intracellular growth, and survival of wild-type* V. cholerae* (dark grey bars) and* OmpA* mutant strains (light grey bars). Zero time is uptake of the bacteria by amoeba cells. Data represent mean ± SD from three different experiments.

To estimate growth and survival of the engulfed bacteria following gentamicin treatment and recultivation, the number of intracellular bacteria was estimated by viable counts. The viable count of uptaken wild-type and* OmpA* mutant of* V. cholerae* was 3.2 × 10^3^ ± 1.6 × 10^3^ cell/mL and 4.0 × 10^3^ ± 5.0 × 10^2^ cell/mL, respectively (Figure 1). The viable counts of intracellular wild-type and* OmpA* mutant of* V. cholerae* after 2 hours were 1.7 × 10^3^ ± 1.2 × 10^3^ cell/mL and 1.4 × 10^3^ ± 2.0 × 10^2^ cell/mL, respectively, while after 24 hours they were 3.0 × 10^5^ ± 1.0 × 10^5^ cell/mL and 1.5 × 10^5^ ± 5.0 × 10^4^ cell/mL, respectively (Figure 1). The uptake and intracellular growth of the wild-type and* OmpA* mutant* V. cholerae* were not significantly different (*t*-test, *P* = 0.68).

### 3.2. Growth and Survival of Wild-Type and* OmpA* Mutant* V. cholerae* Cultivated Alone

**Figure 2:**
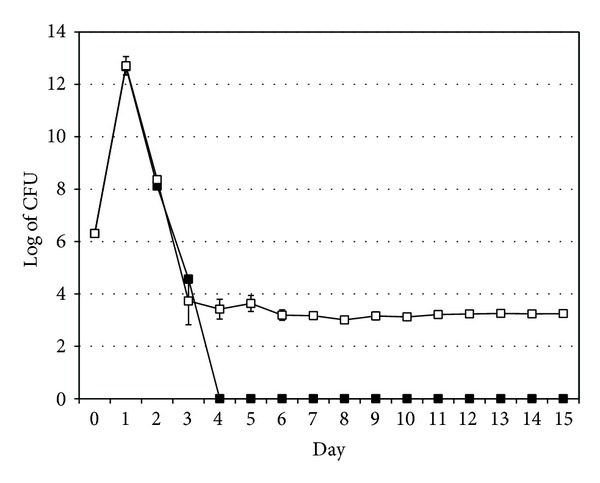
Growth and survival of wild-type* V. cholerae* (filled squares) and the* OmpA* mutant strain (empty squares) cultivated alone. Data represent mean ± SD from three different experiments.

Viable counts of wild-type and* OmpA* mutant* V. cholerae* cultivated alone in the absence of* A. castellanii* showed 10^6^-fold increases after 1 day for both. Surprisingly, the wild-type strain survived for only 3 days, while the* OmpA* mutant of* V. cholerae* survived more than 2 weeks, with a viable count of 1.7 × 10^3^ ± 2.1 × 10^2^ cell/mL at day 15 (Figure 2). The survival rates of the wild-type and* OmpA* mutant of* V. cholerae* were significantly different (*t*-test, *P* = 0.005).

### 3.3. Growth and Survival of Wild-Type and* OmpA* Mutant* V. cholerae* Cocultivated with* A. castellanii*

**Figure 3:**
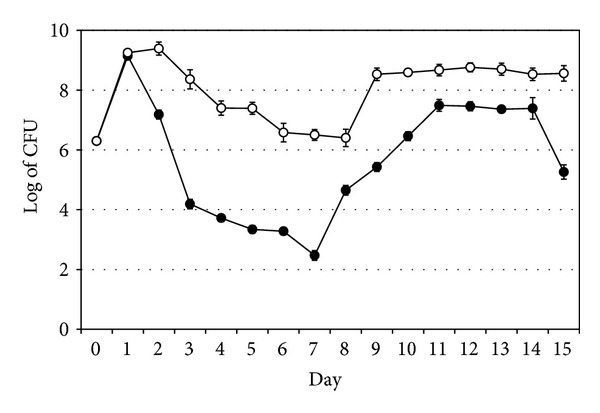
Growth and survival of wild-type* V. cholerae* (filled circles) and the* OmpA* mutant strain (empty circles) when cocultivated with* A. castellanii*. Data represent mean ± SD from three different experiments.

Viable counts of wild-type and* OmpA* mutant* V. cholerae* cocultivated with* A. castellanii* showed 10^3^-fold increases after 1 day for both strains. Surprisingly, both wild-type and* OmpA* mutant* V. cholerae* survived more than 2 weeks, but their viable counts were different (2.0 × 10^5^ ± 1.0 × 10^5^ cell/mL and 4.1 × 10^8^ ± 2.6 × 10^8^ cell/mL at day 15, resp.) (Figure 3).

The presence of* A. castellanii* enhanced survival and growth of both wild-type and* OmpA* mutant stains of* V. cholerae* (Figure 3) compared with that in the absence of* A. castellanii* (Figure 2). Growth of the cocultivated wild-type and* OmpA* mutant strains of* V. cholerae* differed significantly (*t*-test, *P* = 0.0004), with growth of the* OmpA* mutant being higher than that of the wild-type strain (Figure 3).

### 3.4. Growth and Survival of* A. castellanii* Alone or Cocultivated with Wild-Type and* OmpA* Mutant Strains of* V. cholerae*

**Figure 4:**
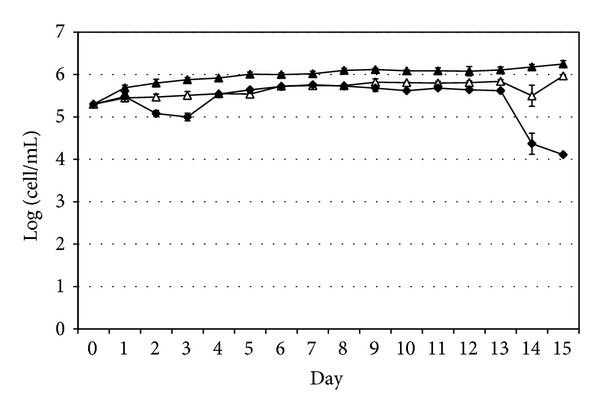
Growth and survival of* A. castellanii* cultivated alone (filled triangles), with wild-type* V. cholerae* (empty triangles) and with the* OmpA* mutant strain of* V. cholerae* (filled diamonds). Data represent mean ± SD from three different experiments.

The number of viable* A. castellanii* alone and cocultivated with wild-type* V. cholerae* increased from 2.0 × 10^5^ ± 0.0 cell/mL to 1.8 × 10^6^ ± 4.2 × 10^5^ and 9.3 × 10^5^ ± 1.8 × 10^5^ cell/mL, respectively, after 15 days. In contrast, the number of viable* A. castellanii* cocultivated with the* OmpA* mutant* V. cholerae* decreased from 2.0 × 10^5^ ± 0.0 cell/mL to 1.3 × 10^4^ ± 1.0 × 10^3^ cell/mL after 15 days (Figure 4).

Cocultivation of the amoebae with* V. cholerae* strains showed that the growth rate of* A. castellanii* alone and in the presence of wild-type or* OmpA* mutant* V. cholerae* was significantly different (*t*-test, *P* = 0.00020 and *P* = 0.00024, resp.). However, the difference between the growth rate of* A. castellanii* cocultivated with wild-type or* OmpA* mutant of* V. cholerae* was less significant (*P* = 0.04).

### 3.5. Production of Outer Membrane Vesicles by* V. cholerae* Strains and Effect of OMVs on* A. castellanii* Viability

**Figure 5:**
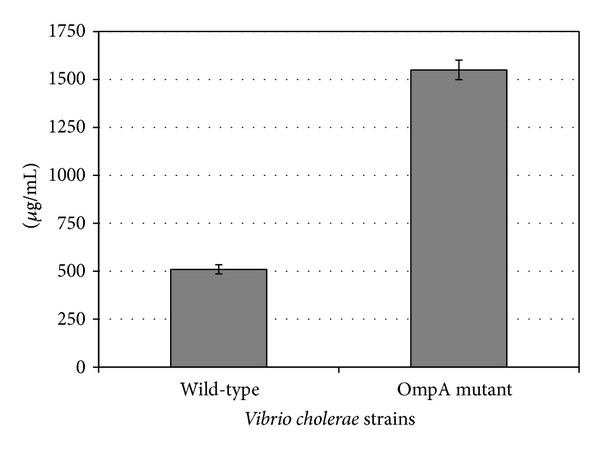
Production of outer membrane vesicles by* V. cholerae* wild-type and* OmpA* mutant strains. Data represent mean ± SD from three different measurements.

OMVs were isolated from the wild-type and* OmpA* mutant strains as described in Section 2. The amount of vesicles released from the two strains was compared by measuring protein concentration, which was found to be 510 ± 24 *μ*g/mL for the wild-type and 1550 ± 51 *μ*g/mL for the* OmpA* mutant strain (Figure 5). This difference in protein concentration was statistically significant (*t*-test, *P* = 0.0001).

**Figure 6:**
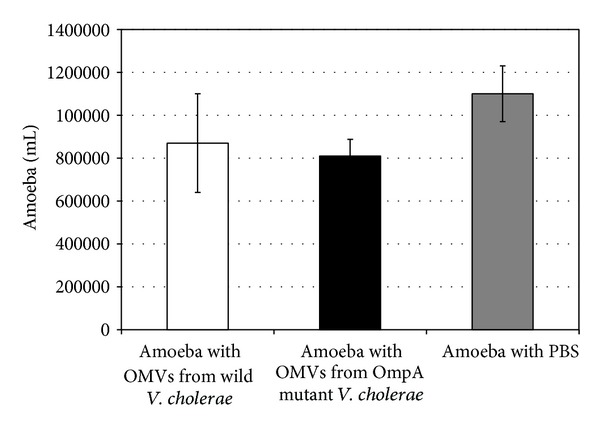
Effect of outer membrane vesicles (OMVs) from wild-type and* OmpA* mutant strains of* V. cholerae* on viability of* A. castellanii*. Data represent mean ± SD from three different measurements.

Treatment of the amoebae with bacterial vesicles showed that the OMVs lowered the viability of the amoeba cells after 2 hours of incubation. Thus the viable count of* A. castellanii* incubated with OMVs from wild-type* V. cholerae*, OMVs from the* OmpA* mutant strain, and PBS was 8.7 × 10^5^ ± 2.3 × 10^5^, 8.1 × 10^5^ ± 7.8 × 10^4^ cell/mL, and 1.1 × 10^6^ ± 1.3 × 10^5^ cell/mL, which represent 79%, 74%, and 100% viability, respectively (Figure 6). The viable count decreased significantly compared with the PBS treatment for the amoebae treated with OMVs from* OmpA* mutant* V. cholerae* (*t*-test, *P* = 0.02), but the decrease was less significant for the amoebae treated with OMVs from wild-type* V. cholerae* (*t*-test, *P* = 0.05). However, this difference in amoeba viability might be due to the ability of the* OmpA* mutant of* V. cholerae* to produce more OMVs, as demonstrated here and by Song et al. [7].

## 4. Discussion

*V. cholerae* utilizes several survival strategies in aquatic environments, such as biofilm formation, switching from smooth to rugose colony morphotypes, and association with free-living amoebae [19]. Studies have shown that* V. cholerae* has enhanced growth in association with* A. castellanii* [1–3] and both microorganisms have been detected in the same water samples from cholera endemic areas [20]. This study investigated the role of OmpA protein and the outer membrane vesicles released by* V. cholerae* in survival and interaction of the bacterium with the eukaryotic host* A. castellanii*.

The results demonstrated that, in the absence of* A. castellanii*, the* OmpA* mutant* V. cholerae* survived for much longer (>15 days) than wild-type* V. cholerae* (3 days). In this context, loss of all CFU from wild-type* V. cholerae* cultivated in this rich medium in the absence of amoebae was observed not only in this paper but also previously for other bacteria such as* Francisella tularensis* [21],* Shigella dysenteriae,* and* S. sonnei* [22].

*Francisella* and* Shigella* species are facultative intracellular bacteria multiplying inside amoeba cells and remaining cultivable during the experiment time compared to the bacteria in absence of the amoebae that became noncultivable. Moreover, interaction of the extracellular bacterium* Pseudomonas aeruginosa* with* A. castellanii* showed that growth and survival of* P. aeruginosa* were the same during the experiment time whether the amoebae were present or absent [23]. However,* V. cholerae* O1,* V. cholerae* O139, and* V. mimicus* lost all CFU from the wild-types cultivated in absence of the amoebae from day 4 of cultivation [22, 24–26] and it was proven that* V. cholerae* died and did not enter the viable but nonculturable (VBNC) state after the loss of all CFU from the wild type [25]. The question is how the* OmpA* mutant of* V. cholerae* survived longer than the wild-type strain.

In the present study, it was found that the* OmpA* mutant of* V. cholerae* produced significantly more OMVs than the wild-type strain, confirming previous findings by Song et al. [7] that the lack of OmpA protein leads to production of more OMVs. An interesting observation in the present study was that significant production of OMVs might have enriched the cultivation medium and supported longer survival of the mutant strain compared with the wild-type* V. cholerae*.

To investigate the effect of OMVs on the amoebae,* A. castellanii* cells were incubated in a suspension of OMVs isolated from each* V. cholerae* strain. The viable counts demonstrated a decreased viability of* A. castellanii* in both cases; this might indicate a virulence role of the OMVs towards the amoebae, in agreement with other studies [7, 27].

Interaction of* V. cholerae* strains with* A. castellanii* involves attachment of bacteria to the amoeba cells, engulfment, intracellular growth, and survival inside the amoebae. The engulfment, intracellular growth, and intracellular survival of the wild-type and* OmpA* mutant* V. cholerae* were not significantly different. In this context, Abd et al. [1] found that the capsule and LPS O-side chain did not affect engulfment, intracellular growth, and intracellular survival of* V. cholerae* O139 when interacted with* A. castellanii*.

The results also showed that the presence of* A. castellanii* enhanced survival of both wild-type and* OmpA* mutant strains of* V. cholerae*. This was in agreement with previous findings that interaction of* A. castellanii* with wild-type* V. cholerae* O139, the capsule mutant strain, and the capsule/LPS double mutant strain enhanced survival of all these bacterial strains [1]. Moreover, in spite of the fact that* V. cholerae* O1 El Tor possesses a mannose-sensitive haemagglutinin fimbria and* V. cholerae* O1 classical does not, they have enhanced survival and their intracellular growth in* A. castellanii* is not significantly different [2]. All these facts may indicate that the intracellular behaviour of* V. cholerae* is a new survival strategy [1, 2].

In this study, the presence of the* OmpA* mutant* V. cholerae* decreased viability of* A. castellanii* significantly more than the wild-type* V. cholerae* did; this might be due to overproduction of OMVs by the mutant strain. The OMVs of* V. cholerae* have been suggested to promote the delivery of virulence factors to bacterial or eukaryotic cells [27]. However, our results showed that OMVs decreased viability of the amoebae, which might indicate that vesicles are a virulence factor.

It has been observed previously that OmpA level is inversely correlated with the amount of OMVs and that the sRNA of* V. cholerae*, which is called vibrio regulatory RNA of* OmpA* (VrrA), increases OMVs production at a rate comparable to the loss of OmpA, since VrrA positively regulates OMVs release through downregulation of OmpA protein [7]. However, inactivation of VrrA resulted in increased colonization of* V. cholerae* in an infant mouse colonization assay. Thus, OmpA protein is important for the colonization of* V. cholerae*, and VrrA RNA may be considered a regulator that modulates the virulence of* V. cholerae* [7].

OMVs formation has been suggested to be linked to turgor pressure of the cell envelope during bacterial growth [28]. Gram-negative bacteria have developed many strategies to enable active virulence factors to gain access to the extracellular environment, typically the tissues or bloodstream of the host organism [29].

Vesicles are the means by which bacteria interact with prokaryotic and eukaryotic cells in their environment. Biochemical analysis and functional characterization of pathogen-derived outer membrane vesicles have demonstrated that this secretory pathway has been taken by pathogens for the transport of active virulence factors into host cells [14]. However, the ability of OMVs to fuse with bacterial membranes and of host cells to deliver content into the cytosol means that these vesicles may be described as bacterial “bombs” for directed intercellular transport of particular bacterial virulence factors into host cells and tissues [14, 30–32]. Further investigations are needed to learn more about the function of the vesicles and their interaction with host cells.

Finely, the results showed that when both strains were cultivated alone, the* OmpA* mutant of* V. cholerae* expressed more OMVs and survived for longer than the wild-type. Moreover, the amount of OMVs isolated from the* OmpA* mutant strain was sufficiently high to decrease viability of amoebae. Cocultivation with* A. castellanii* enhanced survival of both wild-type and* OmpA* mutant strains of* V. cholerae*.

In conclusion, OmpA might have a regulating role in survival of* V. cholerae* since it suppressed its survival while the lack of OmpA enhanced release of OMVs. The OMVs might act as a virulence factor when they supported a long survival of the bacterium and decreased viability of the interacted amoebae.* V. cholerae* might be adapted to survive better in association with eukaryotes.
